# Retrograde Maculopathy or Microcystic Macular Degeneration in Patients With Optic Neuritis due to Neuromyelitis Optica

**DOI:** 10.1155/crop/2502132

**Published:** 2026-07-02

**Authors:** Daniel Kim, Leanne Stunkel

**Affiliations:** ^1^ Saint Louis University School of Medicine, St. Louis, Missouri, USA, slu.edu; ^2^ John F. Hardesty, MD Department of Ophthalmology and Visual Sciences and Department of Neurology, Washington University in St. Louis, St. Louis, Missouri, USA, wustl.edu

**Keywords:** neuromyelitis optica, optic neuropathy, optical coherence tomography, retrograde maculopathy

## Abstract

**Purpose:**

The aim of this study is to highlight findings of retrograde maculopathy (RM) on optical coherence tomography (OCT) as a sequela of neuromyelitis optica associated optic neuropathy.

**Observations:**

We report two patients with demyelinating lesions involving the visual pathways due to neuromyelitis optica in whom RM was seen, and in whom the location of the RM corresponded to their visual deficits. In one case, we describe a patient who had a demyelinating chiasmal lesion with bitemporal hemianopia and who had corresponding binasal RM. In another case, we describe a patient who had generalized vision loss in the right eye and who had corresponding diffuse RM in the right macula.

**Conclusions and Importance:**

RM is a potential sequela of severe optic neuropathy in neuromyelitis optica and topographically corresponds to severe visual field defects, as well as severe ganglion cell complex loss. Providers should recognize this finding and be aware that they do not need to pursue diagnostic evaluation due to concern for primary macular disease.

**Highlights**

• Retrograde maculopathy (RM) may be seen in severe optic neuropathies such as neuromyelitis‐associated optic neuritis.
• RM typically topographically corresponds to visual field loss.
• RM degeneration does not warrant additional diagnostic evaluation or separate treatment.

## 1. Introduction

RM, formerly referred to as microcystic macular edema and also known as microcystic macular degeneration, describes cystic changes in the inner nuclear layer (INL) of the perifoveal retina. These microcystic changes were previously presumed to represent true edema; however, these changes do not exhibit leakage on fluorescein angiography, resulting in an update to the terminology from microcystic macular edema to microcystic macular degeneration [1, 2]. Similar degenerative lesions in the INL have been described in human pathologic studies over 60 years ago, suggesting that this phenomenon has long been recognized [3]. As the pathophysiology of this finding represents a secondary retrograde consequence of optic neuropathy rather than a primary macular degeneration, the term “retrograde maculopathy” has been introduced [4]. RM is typically a sequela of severe optic neuropathy, such as that caused by demyelinating disorders, including neuromyelitis optica spectrum disorder (NMOSD). Additionally, RM tends to be topographically related to the location and severity of vision loss [5–8]. Although RM may appear alarming, it should be considered a secondary manifestation of underlying optic nerve pathology and does not require additional diagnostic workup or direct treatment. Here, we present two patients who presented with optic neuritis due to NMO and who were noted to have RM on OCT corresponding to their vision loss.

## 2. Materials and Methods

Retrospective case series at a tertiary neuro‐ophthalmology clinic, describing two patients with optic neuropathy and RM on OCT.

Patients were included if they had a diagnosis of optic neuropathy and macular INL microcystic changes consistent with RM on spectral‐domain OCT.

From the electronic health record, we extracted demographics, relevant history, symptoms, the best corrected visual acuity, color vision (Ishihara color plates), relative afferent pupillary defect (RAPD), slit‐lamp and fundus findings, confrontational visual field findings. Ophthalmic imaging results were extracted from Humphrey Visual Field Analyzer 24‐2 and Zeiss Cirrus Optical Coherence Tomography. Neuroimaging and serologic testing were recorded when performed. Treatments and follow‐up findings were also noted.

This retrospective study was approved with an informed consent waiver by the Washington University in Saint Louis Institutional Review Board because this chart review involved no patient contact and no identifiers were retained in this manuscript.

## 3. Results (Case Description)

### 3.1. Case 1

A 32‐year‐old woman, who was 34 weeks pregnant and had no other significant medical history, presented to the emergency department for 2 days of oculus sinister (OS) vision loss and pain associated with extraocular movements. Examination showed near visual acuity 20/20 oculus dexter (OD), 20/100 OS, red color desaturation OS (formal color plates were not documented), RAPD OS, and upper temporal quadrant visual field restriction in the OD to confrontation visual field testing. The next morning, OD visual acuity worsened to 20/100 with persistence of the previously noted upper temporal visual field restriction to confrontation, with stable findings OS. MRI of the brain and orbits without contrast (performed without contrast due to pregnancy) showed T2/FLAIR hyperintensity of the left optic nerve and optic chiasm. Additionally, an MRI of the cervical spine without contrast showed a long segment T2/FLAIR hyperintense lesion in the cervical spinal cord, most prominent at the C4 level. Serum testing showed positive aquaporin‐4 antibodies consistent with a diagnosis of neuromyelitis optica (NMO) and she was treated with intravenous methylprednisolone (IVMP) and plasmapheresis (PLEX) while inpatient. Her visual acuity improved to 20/20 OD, 20/200 OS while receiving treatment with IVMP and PLEX, and she was transitioned to oral prednisone and started rituximab infusions.

**Figure 1:**
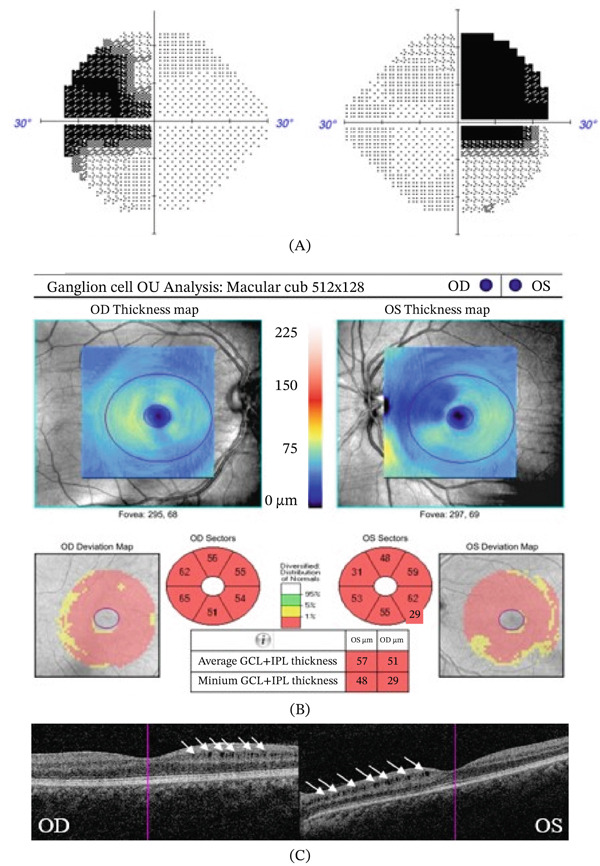
(A) Visual fields: Humphrey visual field 24‐2, performed with Size 3 stimulus in the right eye (shown on the right), Size 5 stimulus in the left eye (shown on the left), showing bitemporal hemianopia, worse superiorly, and generalized depression in the left eye. (B) Optical coherence tomography (OCT) performed on the Zeiss Cirrus showing ganglion cell complex thinning in both eyes, worst binasally in both eyes. (C) Macular OCT of the right eye (shown on the left) and left eye (shown on the right) showing retrograde maculopathy (white arrows) in the nasal hemifield of the macula in each eye.

Two weeks after hospital discharge, she was seen in outpatient follow‐up in our neuro‐ophthalmology clinic. She reported no visual acuity change in the OD and progressive improvement in the OS, with no further eye pain, while on oral prednisone. Examination showed best corrected near visual acuity 20/20 OD, 20/200 OS, Ishihara color plates of 11/11 OD, 3/11 OS, a 1.2‐log unit RAPD OS, and temporal pallor both optic discs, more obvious OS than OD. Humphrey visual field showed a bitemporal hemianopia, worse superiorly, and generalized depression OS (Figure 1A). OCT showed retinal nerve fiber layer (RNFL) thinning bilaterally, worse OS, diffuse ganglion cell complex (GCC) thinning in both eyes (Figure 1B), and RM of the INL in the nasal aspect of the maculae bilaterally (Figure 1C).

### 3.2. Case 2

**Figure 2:**
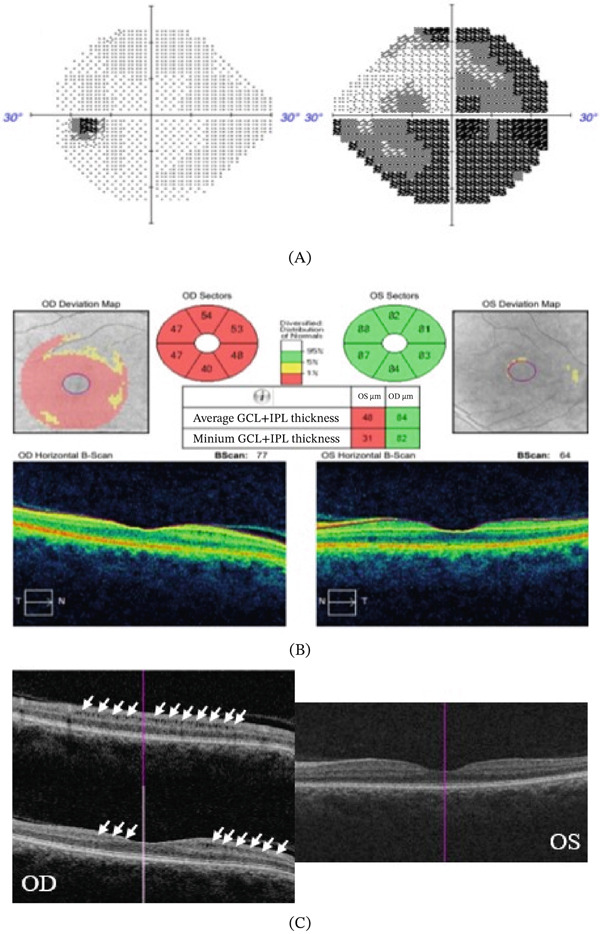
(A) Humphrey visual field 24‐2, performed with Size 5 stimulus in the right eye (shown on the right), Size 3 stimulus in the left eye (shown on the left), showing generalized depression in the right eye and normal findings in the left eye. (B) Optical coherence tomography (OCT) performed on the Zeiss Cirrus, showing diffuse ganglion cell complex thinning in the right eye and normal thickness in the left eye. (C) Macular OCT of the right eye (shown on the left) showing retrograde maculopathy (white arrows) with normal macular OCT in the left eye (shown on the right).

A 42‐year‐old woman with a prior history of severe optic neuritis in the OD several years prior, a history of a longitudinally‐extensive transverse myelitis 1 year following the optic neuritis episode, and well‐controlled hypertension, was referred to neuro‐ophthalmology for evaluation of the persistent visual defect in her OD. The MRI from her optic neuritis episode was not able to be obtained. At the time of the episode of transverse myelitis, an MRI of the brain and pan spine, with and without contrast, had showed a single long‐segment T2 hyperintense, contrast‐enhancing central cord lesion extending from C7–T1 through T3–T4, occupying most of the spinal cord cross‐sectional area. Examination showed the best corrected visual acuity count fingers at 5 feet OD, 20/20 OS, Ishihara color testing showed 0/16 plates OD, 16/16 OS, a 1.2‐log unit RAPD OD, optic nerve pallor OD, and normal optic disc appearance OS. Humphrey visual field testing showed generalized depression OD and full visual field OS (Figure 2A). OCT of the optic nerve displayed abnormal thinning OD (Figure 2B). OCT of the macula displayed RM and diffuse GCC thinning OD and normal OS (Figure 2C). Serum aquaporin‐4 antibody testing was positive, indicating that the underlying etiology of the optic neuritis and transverse myelitis episodes was likely NMO. She started satralizumab (Enspryng) for disease‐modifying therapy.

## 4. Discussion

RM can be seen on OCT in the setting of optic neuropathies from multiple etiologies, including optic neuritis from multiple sclerosis, NMO, and glaucoma [1]. Additionally, the presence of RM on imaging has been noted to correspond to the severity of the optic neuropathy and vision loss [5]. RM has been reported in patients with NMO [1, 5, 6] and noted to be seen more frequently in patients with NMO than in patients with MS [1, 5], possibly due to the severity of NMO‐associated optic neuropathies.

In these two cases, our patient′s initial diagnosis of NMO was confirmed through the presence of autoantibodies against aquaporin‐4, a water protein channel found on astrocytes commonly found in the optic nerve. The immune‐mediated destruction of these channels leads to astrocyte injury, followed by demyelination of the optic nerve, causing optic neuropathy [7].

Thinning of the GCC and impairment of Muller cell function have been proposed to result in the retrograde degenerative changes in the INL [7]. However, there have been new reports that challenge this mechanism of action and instead, hypothesize a mechanical process. The newly proposed process claims that the microcystic changes arise from the loss of cell volume due to injury of ganglion cells and persistent vitreous traction on Muller cell foot plates, causing structural stress on the INL, particularly in the papillomacular bundle. This model may explain why RM does not occur in all cases of optic neuropathy, and this finding is usually confined to younger patients and specific zones in the retina [9].

Additionally, it is important to be aware that retinal edema can lead to a similar appearing but temporary cystic appearance in the inner retinal layer of the macula, such as has been described due to fluid extravasation from the severe optic disc edema that occur in nonarteritic anterior ischemic optic neuropathy (NAION) [10]. These changes may appear similar but resolve over time as the pathophysiology is a result of tissue edema, not retrograde degeneration [10].

Consistent with prior reports [6], in these cases, the location of RM topographically corresponds to the localization of the causative lesions. For example, the RM in the first patient′s OCT imaging was predominantly seen only in the nasal hemifield of the maculae bilaterally, with sparing of the temporal maculae, which corresponds with the most severe areas of binasal GCC thinning and the bitemporal hemianopia seen on her visual field.

Importantly, although RM is found in 20% of patients diagnosed with optic neuropathy and reflects the severity of optic neuropathy, it does not represent a primary retinal disease [7]. Management focuses on treatment of the underlying cause of the optic neuropathy to prevent the further progression of disease [7]. Our report highlights the importance of recognizing RM on OCT as sequelae of severe optic neuropathy.

## Funding

This study was supported by the Research to Prevent Blindness (10.13039/100001818).

## Disclosure

All authors attest that they meet the current ICMJE criteria for authorship.

## Consent

Written informed consent for publication was not obtained. This report was exempted by the institutional review board and all patient information has been deidentified.

## Conflicts of Interest

The authors declare no conflicts of interest.

## Data Availability

The data that support the findings of this study are available on request from the corresponding author. The data are not publicly available due to privacy or ethical restrictions.
